# Chemical and Volatile Compounds in Sweet Potato Brandy: Impact of Processing Methods

**DOI:** 10.3390/foods14091467

**Published:** 2025-04-23

**Authors:** Yunying Li, Lin Li, Qian Liu, Yina Yin, Lin Zhou, Xinxin Zhao, Xinyan Peng

**Affiliations:** 1 College of Life Sciences, Yantai University, Yantai 264005, China; 18596200343@163.com (Y.L.); 15266231782@163.com (Q.L.); yyna0101@163.com (Y.Y.); 18640641841@163.com (L.Z.); 2 Yantai Food and Drug Inspection and Testing Center, Yantai 264000, China; 13905352916@163.com; 3 College of Food Science and Engineering, Yangzhou University, Yangzhou 225127, China; xxzhao@yzu.edu.cn

**Keywords:** sweet potato brandy, volatile organic compounds, gas chromatography–ion mobility spectrometry, processing methods, flavor profile

## Abstract

This study investigated the impact of various thermal processing methods—steaming, boiling, frying, and baking—on the volatile organic compounds (VOCs) in sweet potato (*Ipomoea batatas* L.) brandy using gas chromatography–ion mobility spectrometry (GC-IMS). Yanshu No. 25 sweet potatoes, recognized for their high levels of mucin protein and soluble sugars, were employed for the fermentation of the brandy. GC-IMS analysis generated three-dimensional spectrograms, which revealed distinct VOC profiles depending on the processing method used. Notably, steaming, frying, boiling, and baking significantly altered the VOC composition, imparting unique flavor characteristics. A total of 37 VOCs were identified, with esters being the predominant class, contributing to fruity and floral notes in the brandy. Principal component analysis (PCA) and Euclidean distance-based fingerprint similarity analysis further differentiated the VOC profiles, highlighting the essential role of processing techniques in flavor development. These findings provide a foundation for future research aimed at optimizing processing methods to create specific aromatic profiles in sweet potato brandy.

## 1. Introduction

Sweet potatoes (*Ipomoea batatas* L.), with an annual global production exceeding 90 million tons, represent the sixth most important food crop worldwide and play a crucial role in food security [1]. These tubers are highly nutritious, offering substantial amounts of beta-carotene (7.91 to 12.85 mg/100 g) and essential minerals, such as potassium (260 mg/100 g), phosphorus (51 mg/100 g), and calcium (29 mg/100 g) [2]. Notably, approximately 85% of sweet potato carotenoids are bioavailable, converting into vitamin A, thus providing significant health benefits, particularly in mitigating vitamin A deficiency among children and pregnant women [3]. While traditional preparation methods include steaming, microwaving, baking, and frying [4], there is a growing interest in novel culinary applications, such as fermented sweet potato brandy. This innovative approach leverages the tuber’s inherent sugars and aromatic compounds to enhance flavor complexity during fermentation [5], aligning with contemporary food trends that emphasize healthier and more diverse culinary experiences [6].

In recent years, distilled beverages have garnered increasing attention due to their concentrated aromas and diverse flavor profiles. Unlike fermented beverages, distilled liquors undergo thermal evaporation and condensation, which intensifies volatile compound content and reshapes aroma characteristics. In this context, sweet potatoes offer a promising base material. Orange-fleshed varieties, in particular, provide a unique composition of fermentable sugars and heat-sensitive aroma precursors that are susceptible to transformation during thermal processing [1,3,4]. Studies have shown that cooking methods significantly impact their chemical makeup and sensory-active compounds [1,4], which may further influence the volatile profile of sweet-potato-based beverages. While prior research has explored the nutritional and functional aspects of sweet potatoes in traditional and fermented foods [5,6], their utilization in distilled products remains underexplored. Therefore, understanding how processing affects volatile transformation pathways in sweet potato brandy is both scientifically and practically valuable.

Flavor is a crucial determinant of sensory perception and consumer acceptance in wines [7]. The distinct flavor profiles in wines arise from a complex interplay between non-volatile compounds, such as sugars, acids, and phenolics, and volatile compounds, including esters, higher alcohols, fatty acids, aldehydes, ketones, terpenoids, and volatile phenols [8]. Given the significant role these volatile components play in shaping sensory attributes and overall flavor, their analysis is essential in wine research [9]. However, limited studies have examined the volatile aromatics in fermented sweet potato brandy, particularly those influenced by different cooking methods. To address this gap, the present study employs gas chromatography–ion mobility spectrometry (GC-IMS), a highly sensitive and rapid analytical tool capable of detecting complex volatile profiles across food matrices without requiring prior sample pretreatment [10]. GC-IMS has been widely utilized in food volatile analysis and finds applications in fields like medical diagnostics and environmental monitoring [11]. This technique offers a precise representation of a sample’s flavor profile [12] and has been extensively applied in wine flavor research [13], including studies on yellow wine [14], yellow-fleshed peach wine [7], cherry wine [15], and raspberry wine [16].

This study aims to systematically investigate the volatile organic compounds (VOCs) in sweet potato brandy produced under different processing conditions using GC-IMS. By establishing a comprehensive VOC profile that includes both retention and drift times, we enable detailed comparisons across samples. This analysis will help clarify the impact of processing methods on the volatile composition and sensory qualities of sweet potato brandy. The findings are expected to contribute to a better understanding of flavor formation and provide practical guidance for optimizing processing techniques to enhance product quality and aroma characteristics.

## 2. Materials and Methods

### 2.1. Chemicals and Materials

Yanshu No. 25 sweet potatoes were sourced from Yanda Market (Yantai, Shandong, China), while Arowana brand peanut oil (900 mL) was procured from Zhenhua Supermarket (Yantai, Shandong, China). The fermentation process utilized Wuliangye koji (Niangzhongchun, CZC, Chengdu, Sichuan, China), a commercial fermentation starter produced in accordance with GB26687-2011. According to manufacturer specifications, it is composed of *Saccharomyces cerevisiae*, *Rhizopus* spp., and compound enzyme preparations, and it provides key hydrolytic enzymes, such as amylase, glucoamylase, protease, and lipase, facilitating starch saccharification and protein degradation during fermentation. All chemicals used in the analyses were of analytical grade and purchased from Sinopharm Chemical Reagent Co., Ltd. (Shanghai, China).

### 2.2. Cooking Methods

Yanshu No. 25, or Mishu, is a superior sweet potato variety resulting from sexual hybridization at Yantai Academy of Agricultural Sciences. It is a crossbreed of Lushu No. 3 (female) and Hongrouhong (male). This cultivar boasts superior nutritional values, with 30.2% more mucin protein and 3.48-fold higher soluble sugars per 100 g than common sweet potatoes. Distinctive for its sticky–sweet flavor, moderate β-carotene, and golden yellow flesh, Yanshu No. 25 stands out among standard varieties.

For consistency, Yanshu No. 25 sweet potatoes of uniform size and weight were selected for this study. Fifteen medium-sized tubers (100–150 g) were meticulously washed, air-dried, and weighed before being randomly allocated into five groups. A control group was kept raw (coded as DB1), and the remaining groups were processed via steaming (DB2), boiling (DB3), frying (DB4), or baking (DB5), all without peeling before cooking.

Specifically, DB2 samples were steamed at 100 °C for 40 min using a Midea C30-IH3002 steamer (Midea Group, Foshan, Guangdong, China) [1]; the sweet potatoes were placed on a perforated rack above the boiling water to avoid direct contact with liquid and enclosed in the steamer chamber to ensure exposure to saturated steam only. DB3 samples were boiled in 4 L of boiling water (100 °C) for 30 min in a household stainless-steel pot (SUPOR ST20H1, Supor Co., Ltd., Hangzhou, Zhejiang, China; made of 304-grade stainless steel; diameter 24 cm, depth 11 cm), allowing for full immersion of the sweet potatoes during cooking. DB4 samples were fried in 2 L of oil at 170 °C for 5 min using a Chigo ZG-BK-ZL-81 fryer (Chigo Group, Foshan, Guangdong, China) [17], and DB5 samples were baked at 200 °C for 90 min in a Konkakao-13T1 oven (Konkakao Electrical Appliance Co., Ltd., Zhongshan, Guangdong, China) [1].

### 2.3. Preparation of Ipomoea batatas L. Wine

Following the distinct cooking treatments, the sweet potatoes were cooled to ambient temperature prior to manual peeling. Peeling was performed after thermal processing to minimize peel-derived variability and enhance analytical consistency, particularly in relation to volatile compound profiling. While this step served an experimental purpose, its necessity in industrial contexts may be re-evaluated based on process design and product goals. The peeled flesh was homogenized into a puree using a mixer and then transferred to a fermentation vessel. Pectinase enzyme (0.04 g/L, 100,000 U/g) was added to facilitate the enzymatic breakdown of pectin and improve juice extraction. Although the enzymatic hydrolysis of polysaccharides was not quantitatively monitored prior to sugar adjustment, the soluble solids content (measured as °Brix) was corrected to 25% using exogenous sucrose considering both the native and released soluble constituents. Wuliangye distiller’s yeast (1 g) was evenly distributed across the surface of approximately 350 g of sweet potato mash in each fermentation vessel. Fermentation aimed to yield a final product with an alcohol content of up to 12% (*v*/*v*). The fermentation vessel was sealed and positioned in a well-ventilated, cool environment, with the temperature meticulously maintained at 23 °C. This controlled setting allowed for a natural 20-day fermentation period, during which the sweet potato mash was stirred twice daily to ensure uniform fermentation. Post-fermentation, the mash was distilled using a 5 L laboratory-scale stainless-steel distillation apparatus (Xinrui Instruments Co., Ltd., Yantai, Shandong, China) equipped with a thermometer and condenser, operating under atmospheric pressure. The distillation was conducted as a simple batch process, and the heart fraction was collected between 78 and 95 °C. The fore-shot (first 2% of the distillate) and tail fractions were discarded to eliminate undesirable components. The process was terminated once the alcohol content of the distillate dropped below 25% (*v*/*v*) in order to preserve the volatile aroma compounds and minimize fusel oils. The resulting distilled brandy was then bottled and stored in a cool, dark environment to stabilize before undergoing further analytical evaluation.

### 2.4. Determination of Basic Physicochemical Parameters

The physicochemical analyses were conducted separately for the fermented mash (wine) and the distilled product (brandy) to accurately reflect the characteristics of each stage.

For the fermented grape wine, the pH value, reducing sugars, soluble solids content, and total acidity (expressed as tartaric acid equivalents) were determined according to the slightly modified method of Hao et al. (2024) [18]. The pH was determined using a PHS-3E pH meter (Shanghai INESA Scientific Instrument Co., Ltd., Shanghai, China). Reducing sugars were assessed using the 3,5-dinitrosalicylic acid (DNS) colorimetric method, with glucose as the standard. Soluble solids content (°Brix) was measured with a handheld refractometer (Shanghai INESA Scientific Instrument Co., Ltd., Shanghai, China). Total acidity was determined via acid–base titration and expressed as tartaric acid equivalents (g/L) based on methods modified from Ye et al. (2014) [19] and Jiang et al. (2024) [20].

For the distilled brandy, the alcohol content and total ester content were analyzed. The alcohol content was determined using a DA-130 N portable density meter (Kyoto Electronics Manufacturing Co., Ltd., Tokyo, Japan) by measuring the ethanol volume fraction. Total esters were quantified through saponification of esters in the distillate with standard NaOH solution, followed by titration with standard H_2_SO_4_ solution, and expressed as ethyl acetate equivalents (mg/L).

### 2.5. Sensory Evaluation

The sensory evaluation method for aroma analysis was adapted from the method of Shen et al. (2024) [21], with slight modifications, and conducted in accordance with ISO 8589–2007 standards in the sensory laboratory at Yantai University. The evaluation panel consisted of 10 trained members (5 males and 5 females), aged between 20 and 40 years, recruited from students and faculty in the field of food science at Yantai University. The sensory evaluation protocol was ethically approved by the Yantai University Ethics Committee, and all panelists provided informed consent. Prior to the experiment, each panelist underwent 8 h of training, divided into four 2 h sessions over a two-week period. The training sessions covered sensory attributes of sweet potato brandy, including color, aroma, taste, and typicity, adapted from established sensory standards for distilled beverages. The maximum possible score was 100, and the average score from the panel was used as the final sensory evaluation result. The sensory scoring criteria for sweet potato brandy are outlined in Table 1. To ensure the reliability and accuracy of the sensory descriptive scores, descriptive analysis tests were conducted three times over the course of one week, with each session lasting 60 min. Panelists were given breaks between the evaluation of four different samples, with mouth rinsing using water to reset their senses. Each sample was evaluated in triplicate.

**Table 1:** Sensory evaluation standard of sweet potato brandy.

Evaluation Indicators	Fraction	Evaluation Criteria
Color (20 points)	16~20	Clear, high gloss, transparent, golden yellow
	11~15	Clear, slightly dull, golden yellow, no suspended matter
	6~10	Clear, glossless, light yellow, no obvious suspended matter
	0~5	Turbid, dull, dark yellow, with suspended matter
Aroma (30 points)	25~30	Strong sweet potato aroma, prominent main aroma, rich aroma, long aftertaste
	20~25	More obvious sweet potato aroma, natural and harmonious aroma
	10~20	Light sweet potato fragrance, no odor
	0~10	Lack of fragrance, peculiar smell
Taste (40 points)	30~40	The brandy is full-bodied, mellow, and palatable, with harmonious flavor and lingering aftertaste
	20~30	The taste is harmonious and pure, without any impurities
	10~20	The taste is light and not strong
	0~10	Light brandy, unbalanced taste
Typicality (10 points)	9~10	Typical perfection, unique style
	6~8	Typical and clear, good style
	3~5	Typical, general style
	0~2	The typicality of the brandy is not obvious

### 2.6. Analytical Methods

The GC-IMS analysis of the brandy samples followed the protocol established by Zhang et al. (2023) [22]. A Flavorspec^®^ system (GAS Instrument, Dortmund, Germany) was used, equipped with an MXT-WAX capillary column (30 m × 0.53 mm × 1 μm, Restek, Bellefonte, PA, USA). Each brandy sample (1.0 mL) was placed into a 20 mL headspace vial, sealed with a magnetic screw cap, and incubated at 60 °C for 15 min at 500 rpm to equilibrate the VOCs in the headspace.

After incubation, a 100 μL headspace sample was aspirated into an 85 °C preheated syringe and injected into the GC-IMS system in splitless mode. The column and drift tube were maintained at 80 °C and 45 °C, respectively. The drift gas flow rate was set at 150 mL/min throughout the analysis. High-purity nitrogen (99.999%) served as the carrier gas, with a flow rate program starting at 2 mL/min for the first 2 min, increasing to 10 mL/min for the next 8 min, and followed by a ramp up to 100 mL/min for the subsequent 10 min. The flow rate was then held at 100 mL/min for the remainder of the 30 min analysis.

### 2.7. Statistical Analysis

Three parallel tests were performed on each sample, with the results expressed as mean ± SD. Data were analyzed using IBM SPSS Statistics 26 software, applying one-way ANOVA followed by Duncan’s multiple range test to identify significant differences between treatment groups (*p* < 0.05). Additional analyses included orthogonal partial least squares discriminant analysis (OPLS-DA) using SIMCA-P 14.1 software, with graphs generated via Origin 2021 software.

The GC-IMS system (Flavorspec^®^, GAS Instrument, Dortmund, Germany), equipped with VOCal and three specialized plugins, provides a robust platform for sample analysis. VOCal, as the primary tool, handles both qualitative and quantitative spectral interpretation, utilizing the NIST and IMS databases for substance identification while allowing for database expansion with external standards. Its graphical interface links each data point to specific volatile organic compounds (VOCs), and proper calibration ensures accurate quantification of volatile profiles in brandy samples.

The system’s functionality is enhanced by the Reporter plugin, enabling detailed comparative spectral analysis via 2D and 3D spectra and differential formats. The Gallery Plot plugin supports VOC fingerprinting, offering both qualitative and quantitative perspectives on sample variation. The dynamic principal component analysis (PCA) plugin clusters samples through dynamic PCA, helping to identify unknown compounds.

Additionally, the “nearest neighbor” analysis feature employs a Euclidean distance matrix to improve the accuracy of comparative analysis by distinguishing closely related samples from more distant ones.

## 3. Results and Discussion

### 3.1. Analysis of Main Components of Fermented Sweet Potato Wine and Distilled Brandy

#### 3.1.1. Physicochemical Characteristics of Fermented Sweet Potato Wine

Table 2 illustrates the changes in the major components of fermented sweet potato wine produced under different treatment methods. Soluble solids content, a critical indicator reflecting sugar availability for yeast metabolism, is closely linked to consumer taste preferences [23].

The soluble solids content varied across treatments, with DB1 having the highest value (10.5 ± 0.10%) and DB5 the lowest (9.3 ± 0.09%). This suggests that heat treatments, such as steaming, boiling, frying, and roasting, may reduce the availability of fermentable sugars for yeast metabolism, leading to lower sugar levels in these samples. The reduction in soluble solids, particularly in the DB5 and DB4 samples, could be attributed to the Maillard reaction and sugar caramelization, which diminished the availability of fermentable sugars [24].

Reducing sugars, key indicators of residual sweetness, also varied considerably among treatments. The boiled sample (DB2) had the lowest reducing sugar content (2.4 ± 0.06 g/L), possibly due to sugar leaching into the boiling medium [25]. Unlike moist heat treatments, such as boiling, dry roasting does not promote sugar leaching, potentially allowing for greater sugar preservation in the final product.

Total acidity and pH are also important parameters for the fermentation medium [26,27,28]. The total acidity of the fermented sweet potato wine ranged from 3.9 to 4.5 g/L (expressed as tartaric acid equivalents), with DB1 exhibiting the highest acidity and DB5 the lowest. Slight variations in pH were observed, with DB1 having the lowest pH (3.6 ± 0.05) and DB5 the highest (3.9 ± 0.05).

The lower pH in DB1 could be due to the greater retention of organic acids in raw sweet potatoes, while the higher pH in DB5 might result from the thermal degradation of organic acids during roasting, causing slight alkalinization of the matrix [29].

Lower acidity can help preserve fruity aromas, balance sweetness and acidity, and contribute to a pleasant mouthfeel. However, excessively low acidity may adversely affect the taste of fermented sweet potato wine, leading to sour or bitter notes [30].

Importantly, the acidity levels of the fermented sweet potato wine in this study align with the findings of Zhang et al. (2017) [31], falling within a range conducive to a balanced mouthfeel.

#### 3.1.2. Physicochemical Characteristics of Distilled Sweet Potato Brandy

After distillation, only volatile components, such as ethanol and esters, are retained in the final brandy, whereas non-volatile substances, like reducing sugars, soluble solids, and most acids, are eliminated during the distillation process. Thus, only alcohol content and total ester concentration are meaningful indicators for the distilled product.

Alcohol content, a critical parameter in fermented fruit brandy, plays a pivotal role in the traditional alcoholic fermentation process [32].

The alcohol content varied across treatments, ranging from 22.00% (DB5) to 24.50% (DB1), with DB1 exhibiting the highest concentration. This suggests that differences in fermentable sugar availability at the wine stage influenced the final ethanol yield in brandy samples.

Esters, major contributors to brandy aroma and flavor, varied significantly across the samples. DB1 contained the highest ester content (480 ± 5.00 mg/L), while DB5 exhibited the lowest (430 ± 3.50 mg/L). The elevated ester levels in DB1 suggest a more active esterification process, likely due to the retention of volatile precursors in raw sweet potatoes. In contrast, the lower ester levels in heat-treated samples, especially DB5, may result from thermal degradation of ester precursors or their conversion into non-volatile compounds during high-temperature processing [33].

As further detailed in the following sections, the analysis of sweet potato brandy subjected to different thermal treatments underscores the crucial role of processing in determining the chemical composition and sensory properties of the final product. Brandy produced from raw sweet potatoes retained more volatile precursors, resulting in higher alcohol content and a richer ester profile. In contrast, roasted and fried sweet potato brandy showed lower ester and alcohol levels, contributing to different flavor profiles. These findings offer valuable insights for the targeted modulation of sweet potato brandy characteristics through controlled processing conditions, aiding in the development of high-quality, diverse sweet potato alcoholic beverages.

**Table 2:** Main physicochemical characteristics of sweet potato wine and brandy derived from different thermal pretreatments.

	Alcohol Content (%Vol, Brandy)	Soluble Solids Content (%, Wine)	Total Esters (mg/L, Brandy)	Reducing Sugars (g/L, Wine)	Total Acid (g/L, Wine)	pH Value (Wine)
DB1	24.50 ± 0.15 a	10.5 ± 0.10 a	480 ± 5.00 a	3.8 ± 0.05 b	4.5 ± 0.05 a	3.6 ± 0.05 a
DB2	22.80 ± 0.12 b	9.8 ± 0.08 b	450 ± 4.00 b	4.2 ± 0.06 a	4.0 ± 0.03 b	3.7 ± 0.04 a
DB3	24.30 ± 0.13 a	10.3 ± 0.12 a	475 ± 5.00 a	4.0 ± 0.04 a	4.4 ± 0.04 a	3.6 ± 0.05 a
DB4	22.50 ± 0.10 b	9.5 ± 0.07 b	440 ± 3.00 b	4.1 ± 0.05 a	4.0 ± 0.03 b	3.8 ± 0.04 a
DB5	22.00 ± 0.12 c	9.3 ± 0.09 b	430 ± 3.50 c	4.3 ± 0.06 a	3.9 ± 0.03 b	3.9 ± 0.05 a

**Table 2**: Wine-related parameters (pH, total acidity, soluble solids, reducing sugars) were measured before distillation. Brandy-related parameters (alcohol content, total esters) were measured after distillation. Significant differences between the samples are denoted by letters (a–c). Total acidity is expressed in g/L as tartaric acid equivalents. DB1: control group; brandy made from raw sweet potatoes. DB2: brandy made from steamed sweet potatoes. DB3: brandy made from boiled sweet potatoes. DB4: brandy made from fried sweet potatoes. DB5: brandy made from baked sweet potatoes.

### 3.2. Sensory Evaluation

**Figure 1:**
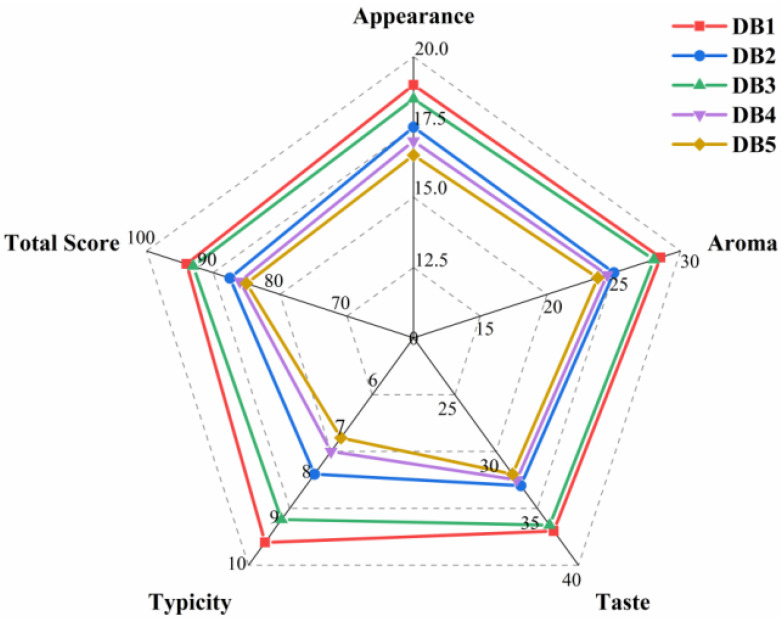
Radar chart of sensory evaluation of brandy fermented with sweet potatoes after different treatments. DB1: control group; brandy made from raw sweet potatoes. DB2: brandy made from steamed sweet potatoes. DB3: brandy made from boiled sweet potatoes. DB4: brandy made from fried sweet potatoes. DB5: brandy made from baked sweet potatoes.

A sensory evaluation of sweet potato brandy fermented with different processing methods revealed significant differences across multiple sensory dimensions (appearance, aroma, taste, and typicity), ultimately affecting the overall scores (Figure 1). The sensory evaluation was conducted on the final distilled product (i.e., sweet potato brandy), not the fermented mash (wine). In terms of appearance, sample DB1 received the highest score (19 points) (Figure 1), indicating that the unprocessed sweet potato retained an optimal color and clarity compared to the heat-treated samples. In contrast, DB5 had the lowest appearance score (16.5) (Figure 1), likely due to subtle changes in clarity or gloss perceived by panelists, which may have resulted from the thermal treatment of the raw material prior to fermentation and distillation [24].

Aroma scores displayed a clear trend, with DB1 scoring the highest (28.5) and DB5 the lowest (23.8) (Figure 1). The high aroma score for DB1 can be attributed to the preservation of volatile compounds, such as esters and aldehydes, which are better retained in raw sweet potatoes (Figure 6). Heat treatments, especially roasting and frying, tend to reduce the concentration of these volatile compounds, thereby diminishing the aroma characteristics.

In terms of taste, DB3 outperformed the other samples with a score of 36.5 points (Figure 1). Steaming, as a moist heat method, may enhance the sweetness and flavor retention of sweet potatoes [34] while minimizing the degradation of essential flavor compounds [35]. In contrast, DB5 and DB2 received significantly lower taste scores, likely due to the breakdown or loss of delicate flavor compounds during more intense heat treatments.

For typicity, DB1 again achieved the highest score (9.5) (Figure 1), emphasizing that the unprocessed sweet potato brandy retained the most recognizable sensory attributes of the raw material. As expected, DB5 scored the lowest (7.2) (Figure 1), likely due to the substantial changes introduced by roasting, which masked the original sweet potato flavors.

Overall, DB1 (94 points) and DB3 (93 points) achieved the highest total scores (Figure 1), highlighting the importance of minimal or moderate processing in preserving the sensory quality of sweet potato brandy. In contrast, DB5 (85 points) had the lowest total score (Figure 1), indicating that although roasting may introduce some complex flavors, it may not align with consumer preferences for traditional sensory attributes in sweet potato brandy.

These findings are consistent with Figure 6, where DB1 retained the highest levels of esters, alcohols, and aldehydes, contributing to its excellent sensory characteristics. The significant differences between DB1 and the heat-treated samples (especially DB5) underscore the critical role of processing methods in shaping the flavor and aroma profiles of fermented sweet potato products. This suggests that while heat treatments, such as roasting, may introduce complex flavors, they may result in a decline in overall sensory quality, particularly for beverages like brandy, where the preservation of the raw material’s inherent characteristics is highly valued.

These sensory differences are strongly supported by the chemical data presented in Table 2 and Table 3. For example, DB1’s high aroma and typicity scores align with its elevated total ester concentration (480 mg/L) and the presence of key fruity volatiles, such as ethyl acetate and isoamyl acetate, as revealed in the GC-IMS analysis. The superior taste score of DB3 may be attributed to its moderate ester retention and a favorable balance of reducing sugars (4.0 g/L) and acidity (4.4 g/L), which contribute to a rounder and more pleasant mouthfeel. Conversely, the lower aroma and taste scores of DB5 are consistent with its reduced ester content (430 mg/L), increased levels of methanol and ketones, and likely thermal degradation of flavor-active compounds during roasting. These correlations between sensory outcomes and chemical profiles help strengthen the mechanistic understanding of how thermal pretreatments influence both the analytical and perceptual qualities of sweet potato brandy.

### 3.3. GC-IMS Analysis of Volatile Compounds in Fermented Sweet Potato (Ipomoea batatas L.) Brandy Following Diverse Processing Techniques

Using GC-IMS, we analyzed the volatile composition of sweet potato (*Ipomoea batatas*) brandy processed through various methods. GC-IMS offers a sensitive and direct assessment of volatile compound profiles, accurately capturing the aromatic profiles of the brandies without the need for sample enrichment [36]. The analytical basis of GC-IMS relies on differential ion migration times, which effectively resolve VOCs in sweet potato brandy produced through different processing techniques [37].

**Figure 2:**
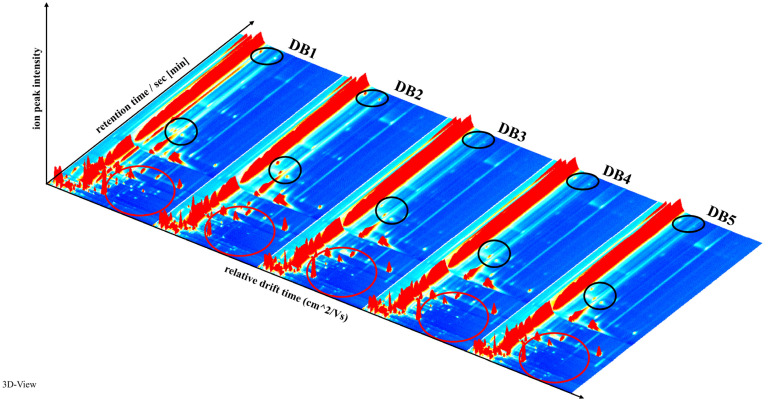
Three-dimensional spectrogram of volatile components in samples. DB1: control group; brandy made from raw sweet potatoes. DB2: brandy made from steamed sweet potatoes. DB3: brandy made from boiled sweet potatoes. DB4: brandy made from fried sweet potatoes. DB5: brandy made from baked sweet potatoes.

The GC-IMS analysis results are presented as 3D spectrograms, shown in Figure 2. The x-axis represents drift time, the y-axis represents retention time, and the z-axis represents peak intensity. These three-dimensional plots clearly demonstrate the differences in VOCs among the brandy samples [38]. Visual inspection of Figure 2 reveals that while the peak signal distributions are generally similar across all samples, subtle yet discernible differences in signal intensity, highlighted by black circles, suggest minor but significant variations in the volatile profiles of the brandies processed in different ways.

A closer examination of the variations marked by red circles in Figure 5 offers further insights into the potential sources of these differences. They may result from a single volatile compound producing multiple signals, either as monomers or dimers, depending on its concentration and chemical properties [39]. Additionally, the formation of adducts between analyzed ions and neutral molecules, such as trimers, could generate multiple signals from a single compound during ion migration, as reflected in the data [40].

Analysis of the red-circled regions in Figure 5 highlights that brandy produced from raw sweet potatoes (DB1) exhibits significant differences from the other samples. This variation is likely due to the retention of more pristine flavor components, such as aldehydes, in the unheated sweet potatoes [41]. The dense signal peaks in Figure 5 confirm the elevated presence of these native volatiles in the raw sweet potato brandy, emphasizing its aromatic complexity.

**Figure 3:**
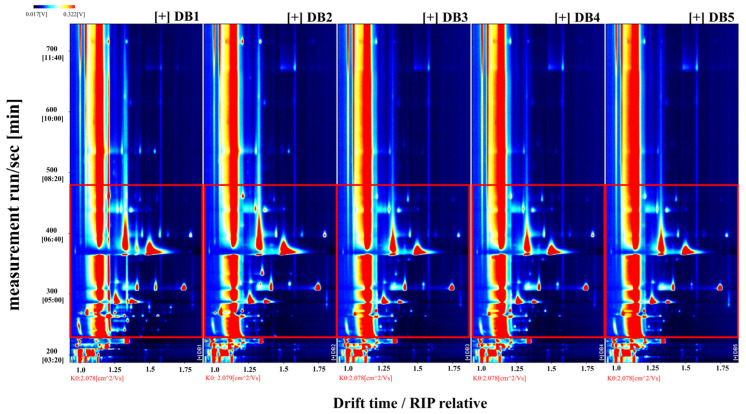
Spectrogram (overhead view) of volatile components in the sample. RIP: representing the reaction ion peak. DB1: control group; brandy made from raw sweet potatoes. DB2: brandy made from steamed sweet potatoes. DB3: brandy made from boiled sweet potatoes. DB4: brandy made from fried sweet potatoes. DB5: brandy made from baked sweet potatoes.

The two-dimensional overhead view in Figure 3 provides further insight into the variation in volatile compounds. The background of the overhead plot is predominantly blue, with a prominent red vertical line at the x-coordinate of 1.0, representing the reaction ion peak (RIP). The RIP serves as a normalization feature, originating from the interaction of water in the headspace with energetic electrons from the tritium (^3^H) ionization source in the IMS, leading to the formation of protonated water clusters. When volatile compounds enter the IMS ionization region, the intensity of the RIP may diminish or disappear, indicating the presence of those compounds [42].

Normalization is critical for standardizing ion drift times and compensating for fluctuations due to temperature and pressure variations during detection [43]. The distinct masses and charges of volatile compounds, combined with their interactions with neutral gas molecules and the electric field within the ion drift region, enable ion separation. This separation is essential for qualitative and quantitative analysis, as it correlates ion drift times with the intensities of ion response peaks, allowing for accurate assessment of volatile compounds [44].

On the display, each point adjacent to the RIP corresponds to a unique volatile organic compound (VOC). The color intensity of these points reflects compound concentration, with white indicating lower levels and red representing higher concentrations, where more intense colors correspond to greater abundance [45]. The y-axis of the plot denotes retention time as determined through gas chromatography, and the x-axis represents the normalized ion drift time.

Figure 3 shows that the majority of signals are concentrated between retention times of 200 and 720 s and drift times of 1.0 and 1.80 ms. The red-framed area for sample DB1 reveals the highest VOC content, demonstrating a pronounced difference from the other samples, consistent with the findings in Figure 2.

**Figure 4:**
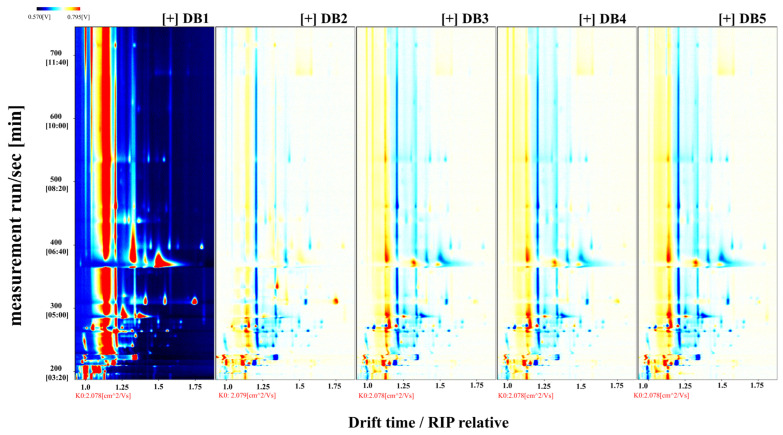
Differential spectrogram comparing volatile component differences in the sample. RIP: representing the reaction ion peak. DB1: control group; brandy made from raw sweet potatoes. DB2: brandy made from steamed sweet potatoes. DB3: brandy made from boiled sweet potatoes. DB4: brandy made from fried sweet potatoes. DB5: brandy made from baked sweet potatoes.

To assess the effects of different processing methods on the VOCs in sweet potato fermented brandy, Figure 4 employs differential contrast mode [46]. Here, the spectral data from sample DB1, as presented in Figure 3, serve as a reference. Differential spectra are generated by comparing the spectral profiles of other samples against this benchmark. A uniform white background in the differential spectrum indicates equivalent VOC levels across samples. Red regions signify areas where VOC concentrations exceed those in the reference sample (DB1), with the intensity of red reflecting the magnitude of the increase. Conversely, deep blue indicates areas where concentrations are lower than in DB1.

Figure 4, in differential contrast mode, reveals substantial differences between samples DB1 and DB2, potentially due to the effects of boiling—a moist heat process that promotes non-enzymatic browning and decomposition in sweet potatoes [47]. Boiling is known to inactivate enzymes, such as lipase, thereby enhancing flavor complexity. Notably, the VOC concentration in DB2, particularly between retention times of 200 and 305 s and drift times of 1.0 and 1.2 ms, surpasses that in DB1. This suggests that boiling may liberate a broader range of volatile compounds, including monoterpenes, benzene derivatives, and furans, contributing to the brandy’s aroma profile [1].

The differential spectrum for DB2 also shows extensive regions of intense blue between retention times of 210 and 330 s and drift times of 1.2 and 1.65 ms, indicating significant differences in VOC concentrations compared to DB1. This variation could result from mechanical stress during boiling, which can induce the evaporation or decomposition of VOCs [48]. Consistent with this observation, a reduction in certain VOC concentrations has been documented in boiled millet [49]. At drift times of 1.35 ms and 1.75 ms, DB2 displays two prominent red points, suggesting elevated concentrations of sesquiterpenes and possibly ketones or maltol, an aromatic compound characteristic of boiled sweet potatoes [50,51].

Analysis of samples DB3, DB4, and DB5—representing steamed, fried, and baked sweet potato brandy, respectively—reveals analogous variations in their VOC profiles. These differences are commonly associated with thermal processing and the Maillard reaction, a non-enzymatic browning process in which carbonyl compounds react with amino acids to generate unique flavor compounds at elevated temperatures [52]. When comparing DB1 and DB5, the differential spectrum, particularly within drift times of 1.0–1.7 ms, exhibits a spectrum of red and blue points, indicating a range of concentration disparities. The characteristic flavors in baked sweet potatoes are attributed to pyrolytic processes, including the release of terpene glycosides, the degradation of carotenoids, caramelization, Maillard reactions, and Strecker degradation [53]. These reactions may enhance volatility or promote the accumulation of specific compounds during baking, leading to the observed changes in VOC concentrations in DB5 [54].

The marked divergence between DB2 and DB5 within retention times of 305–550 s and drift times of 1.3–1.75 ms highlights the enhanced aromatic profile of baked sweet potatoes relative to boiled ones, indicating distinct flavor outcomes [55].

### 3.4. Characterization of Volatile Compounds in Sweet Potato Fermented Brandy Processed via Diverse Methodologies

**Figure 5:**
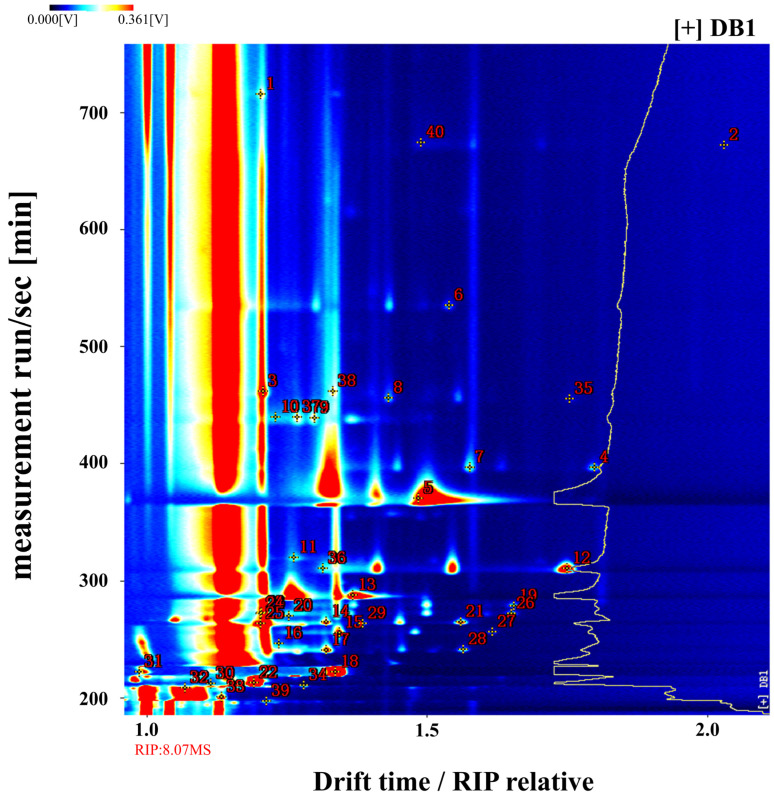
Qualitative analysis using library search. RIP: representing the reaction ion peak; DB1: control group; brandy made from raw sweet potatoes.

In this investigation, volatile compound identification was conducted by comparing IMS drift times and retention indices with those of authentic standards by utilizing the NIST and IMS databases integrated into the GC-IMS system [56]. A qualitative analysis of the volatile constituents in raw sweet potato fermented brandy samples was performed, producing detailed profiles of the identified compounds. As shown in Figure 5, the x-axis represents differential time and the y-axis indicates resolution time, with the corresponding compounds marked by red numerals and listed in Table 3.

Although the databases provided extensive coverage, some limitations resulted in three compounds remaining unidentified [57]. Table 3 lists the compounds depicted in Figure 5, with esters highlighted as the most abundant, contributing significantly to the brandy’s fruity and floral notes, thereby enriching the flavor profile of the sweet potatoes [58]. The VOCs in the Yanshu No. 25 sweet potato fermented brandy were predominantly esters. The analysis revealed 37 distinct volatile compounds across 40 detected peaks, consisting of 22 esters, 7 alcohols, 4 ketones, 2 aldehydes, 1 terpene, and 1 alkylbenzene, showcasing the varietal’s distinctive volatile profile [59].

In the drift region, multiple signals for individual compounds were observed, likely due to the formation of adducts between analyzed ions and neutral molecules, including dimers [60]. Compounds with high proton affinity may form dimers or trimers during their transit through the ion drift tube, a process influenced by their concentration and half-lives within the tube [61]. As noted by Garrido-Delgado et al. (2015) [62], the presence of monomers and dimers depends on the concentration and chemical stability of volatile substances, highlighting the complexity of ion behavior in the drift region.

Eight volatile compounds were identified in both monomeric and dimeric forms: ethyl octanoate-D, isoamyl acetate-D, ethyl 2-methylbutanoate-M, ethyl butanoate-D, ethyl butanoate-M, ethyl 2-methylbutanoate-D, isoamyl acetate-M, and ethyl octanoate-M. This aligns with the findings of He et al. (2023) [63], who emphasized the significant contribution of esters and alcohols to the aroma profile of spirits, corroborating the outcomes of our analysis.

**Table 3:** List of compounds.

Count	Compound	CAS	Formula	^a^ MW	^b^ RI	^c^ Rt [sec]	^d^ Dt [a.u.]
1	2-butoxyethanol	111762	C_6_H_14_O_2_	118.2	1453.6	715.276	1.20492
2	Ethyl octanoate-D	106321	C_10_H_20_O_2_	172.3	1430.7	671.837	2.03135
3	1,3-diethylbenzene	141935	C_10_H_14_	134.2	1293.3	461.885	1.20966
4	Ethyl hexanoate	123660	C_8_H_16_O_2_	144.2	1232.4	397.007	1.80067
5	3-Methyl-1-butanol	123513	C_5_H_12_O	88.1	1204.8	370.638	1.48651
6	Pentyl hexanoate	540078	C_11_H_22_O_2_	186.3	1347.5	535.298	1.54056
7	Limonene	138863	C_10_H_16_	136.2	1232.4	396.955	1.5777
8	3-Methylbutyl 2-methylbutanoate	27625350	C_10_H_20_O_2_	172.3	1288.1	455.962	1.43329
9	3-Octanone	106683	C_8_H_16_O	128.2	1272.6	438.713	1.30156
10	1-hydroxy-2-propanone	116096	C_3_H_6_O_2_	74.1	1273.1	439.206	1.23163
11	ethyl pentanoate	539822	C_7_H_14_O_2_	130.2	1138.6	319.94	1.26416
12	Isoamyl acetate-D	123922	C_7_H_14_O_2_	130.2	1124.9	310.576	1.75203
13	2-Methylpropanol	78831	C_4_H_10_O	74.1	1086.6	287.413	1.36986
14	Propyl propanoate	106365	C_6_H_12_O_2_	116.2	1036.6	264.63	1.32208
15	2-butanol	78922	C_4_H_10_O	74.1	1016.3	255.901	1.34564
16	isobutyl acetate	110190	C_6_H_12_O_2_	116.2	992.6	246.58	1.23794
17	Ethyl propanoate	105373	C_5_H_10_O_2_	102.1	969.5	240.81	1.32208
18	Ethyl Acetate	141786	C_4_H_8_O_2_	88.1	889.2	221.725	1.33779
19	ethyl 3-methylbutanoate	108645	C_7_H_14_O_2_	130.2	1067.9	278.685	1.65689
20	ethyl 2-methylbutanoate-M	7452791	C_7_H_14_O_2_	130.2	1048.7	269.956	1.25545
21	Ethyl butanoate-D	105544	C_6_H_12_O_2_	116.2	1037	264.778	1.56217
22	Methyl acetate	79209	C_3_H_6_O_2_	74.1	848.7	212.7	1.19344
23	(E)-2-butenal	123739	C_4_H_6_O	70.1	1053.8	272.274	1.2053
24	Ethyl butanoate-M	105544	C_6_H_12_O_2_	116.2	1034	263.477	1.20435
25	ethyl 2-methylbutanoate-D	7452791	C_7_H_14_O_2_	130.2	1053.6	272.138	1.65246
26	pentyl formate	638493	C_6_H_12_O_2_	116.2	1017.3	256.304	1.61821
27	Ethyl 2-methy lpropionate	97621	C_6_H_12_O_2_	116.2	970.9	241.147	1.56588
28	1	unidentified	*	0	1034.6	263.748	1.38702
29	Acetone	67641	C_3_H_6_O	58.1	844.5	211.805	1.11664
30	Methanol	67561	CH_4_O	32	891.9	222.355	0.98861
31	Ethyl formate	109944	C_3_H_6_O_2_	74.1	828.6	208.358	1.06969
32	2	unidentified	*	0	792.1	200.692	1.13492
33	Butanal	123728	C_4_H_8_O	72.1	838.2	210.422	1.28146
34	2-Octanone	111137	C_8_H_16_O	128.2	1287.2	454.973	1.75587
35	Isoamyl acetate-M	123922	C_7_H_14_O_2_	130.2	1124.2	310.119	1.31645
36	1-pentanol	71410	C_5_H_12_O	88.1	1273.4	439.568	1.27064
37	3-Hydroxy-2-butanone	513860	C_4_H_8_O_2_	88.1	1293.1	461.642	1.33433
38	3	unidentified	*	0	774.4	197.071	1.21516
39	Ethyl octanoate-M	106321	C_10_H_20_O_2_	172.3	1431.9	674.043	1.49085
40	Hexyl acetate	142927	C_8_H_16_O_2_	144.2	1272.1	438.223	1.36447

**Table 3**: ^a^ MW: Molecular Weight; ^b^ RI: Retention Index; ^c^ Rt [sec]: retention time; ^d^ Dt [a.u.]: drift time; The “*” indicates compounds that were not detected.

### 3.5. Comparative Spectroscopic Analysis of Fermented Brandy Derived from Sweet Potatoes Processed via Diverse Methods

**Figure 6:**
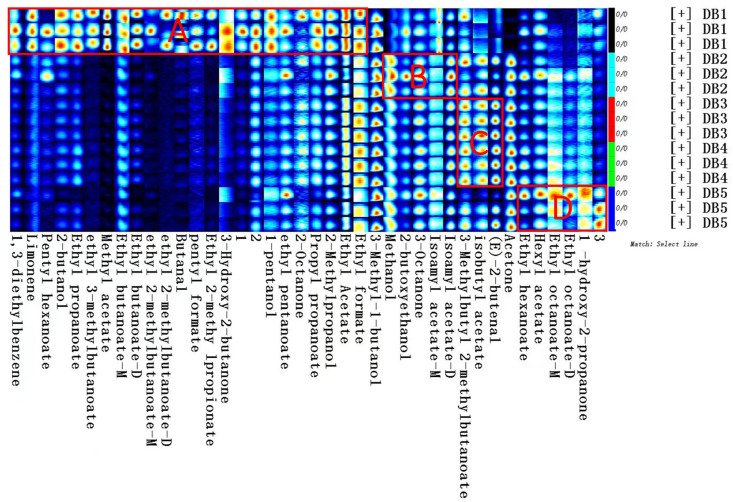
Fingerprint spectrogram of volatile compounds in fermented sweet potato brandy. DB1: control group; brandy made from raw sweet potatoes. DB2: brandy made from steamed sweet potatoes. DB3: brandy made from boiled sweet potatoes. DB4: brandy made from fried sweet potatoes. DB5: brandy made from baked sweet potatoes. Areas A–D correspond to characteristic regions enriched with key esters contributing to fruity and floral aromas.

This study employs fingerprint analysis to address the limitations of assessing individual volatile flavor compounds in sweet potato brandy, as traditional morphology charts and ion mobility spectra provide only a broad overview of compound composition and concentration [40]. Fingerprint spectra offer a clear visualization of the variations in volatile components among brandy samples processed using different methods [64], as illustrated in Figure 6. Each row represents the signal peaks within an individual sample, and each column shows the signal intensity for a specific volatile compound across different samples. The intensity of each dot corresponds to the concentration of the compound, with more vivid colors indicating higher concentrations. Numbers in Figure 6 correspond to substances that have yet to be characterized in the migration spectra library.

Figure 6 reveals that sample DB1 contains the greatest diversity and concentration of VOCs. While samples DB2 through DB5 display fewer volatile substances, they each exhibit unique flavor profiles attributed to their respective processing methods. Raw sweet potato brandy (DB1) is dominated by esters, alcohols, and ketones, complemented by aldehydes, terpenes, and alkylbenzenes. Boiled sweet potato brandy (DB2) also features esters, alcohols, and ketones, while steamed and fried sweet potato brandies show a predominance of esters and aldehydes. In contrast, baked sweet potato brandy (DB5) is characterized by a higher concentration of esters and ketones, with significant levels of methanol and acetone contributing to flavor enhancement post-processing.

Esters, prevalent in all processed samples, play a pivotal role in contributing fruity sensory attributes to the brandy [65,66]. In DB1, esters, such as isoamyl acetate and ethyl acetate, impart winy and sweet fruity aromas (Table 4), respectively (Area A) [67]. Ethyl butyrate adds apple and pineapple notes to DB1 [68]. Meanwhile, ethyl formate and ethyl propionate contribute a range of sweet, fruity, vinous, floral, balsamic, and cocoa aromas [18]. These esters suggest that raw sweet potato brandy predominantly exhibits fruity characteristics, with ethyl acetate being a quintessential marker of fruity aromas in spirits [69]. In DB2, isoamyl isobutyrate (Area B) introduces a distinct banana flavor (Table 4), while isovaleric acid ethyl ester (Area C) and ethyl 2-methyl-3-methylbutyrate (Area D) in DB3 and DB4 contribute fruity, apple, banana, and grassy notes (Table 4). DB5 is characterized by high concentrations of esters, like ethyl hexanoate, which imparts pineapple and green nuances [70]. Ethyl octanoate (Area D) in DB2 adds apricot-like aromas [71], while ethyl hexanoate enhances the fruity and herbal notes (Table 4), refining DB2’s aroma and smoothness. The ester composition of DB2 through DB5 differs from DB1, possibly due to heat-induced enzymatic inactivation, which may hinder ester formation [6].

Alcohols, which enhance the complexity of spirits’ aromatic profiles [7], also play an important role in these samples. In DB1, alcohols, such as 2-butanol and isobutanol, impart brandy-like and solvent-like odors (Table 4), while DB2 is characterized by methanol and 2-butoxyethanol, which introduce fruity and herbal nuances (Table 4). In DB5, ethyl acetate methanol adds spicy, sweet, caramel, and ethereal flavors (Table 4). No unique alcohols were detected in DB3 or DB4. A commonality across all samples is the presence of 3-methyl-1-butanol, which imparts whiskey, malt, and charred flavors to the Yanshu No. 25 sweet potato brandy (Table 4).

Aldehydes and ketones are key to enhancing brandy’s complexity and modulating aromatic compound release [72]. In DB1, butanal contributes spicy, green scents, 3-hydroxy-2-butanone imparts buttery and creamy notes, and 2-octanone introduces soapy, gasoline-like flavors (Table 4). In DB2, 3-octanone contributes distinctive vanilla, buttery, and resinous flavors (Table 4). Trans-2-butenal in DB3 and DB4 provides floral aromas (Table 4). Interestingly, DB5 lacks distinctive aldehydic and ketonic compounds, although acetone—absent in DB1 but present in DB2, DB3, DB4, and DB5—imparts a refreshing fruity aroma (Table 4). This variation in aldehydes and ketones across samples may result from differences in free amino acids, which serve as precursors for aldehyde formation via Strecker degradation [73].

In summary, comparing the identified compounds listed in Table 3 with the fingerprint spectra in Figure 6 reveals significant differences in the volatile profiles, particularly between DB1 and the other samples. DB1 displays the most pronounced concentration of compounds, such as 3-hydroxy-2-butanone, ethyl acetate, and several esters, including ethyl 2-methylpropionate, ethyl 2-methylbutyrate, ethyl 3-methylbutyrate, ethyl butyrate, ethyl hexanoate, ethyl pentanoate, and ethyl formate. DB5, however, is distinguished by higher levels of hydroxyacetone and ethyl octanoate, while ethylene glycol monobutyl ether, 3-octanone, and isoamyl acetate are predominantly found in DB2. The minimal differences between DB3 and DB4 suggest similar processing effects on the volatile composition of these samples.

**Table 4:** Components and odor characteristics of volatile compounds identified in samples through GC-IMS.

IMS Code	Compound	CAS^#^	Formula	^a^ RI	^b^ Rt [sec]	^c^ Dt [a.u.]	^#^ Odor Characteristics *	^d^ MW
	**Esters**							
2	Ethyl octanoate-D	106321	C_10_H_20_O_2_	1430.7	671.837	2.03135	fruit, fat	172.3
4	Ethyl hexanoate	123660	C_8_H_16_O_2_	1232.4	397.007	1.80067	apple peel, fruit	144.2
6	Pentyl hexanoate	540078	C_11_H_22_O_2_	1347.5	535.298	1.54056	fruity	186.3
8	3-Methylbutyl2-methylbutanoate	27625350	C_10_H_20_O_2_	1288.1	455.962	1.43329	fruit, banana, grass	172.3
11	ethyl pentanoate	539822	C_7_H_14_O_2_	1138.6	319.94	1.26416	yeast, fruit	130.2
12	Isoamyl acetate-D	123922	C_7_H_14_O_2_	1124.9	310.576	1.75203	banana	130.2
14	Propyl propanoate	106365	C_6_H_12_O_2_	1036.6	264.63	1.32208	pineapple	116.2
16	isobutyl acetate	110190	C_6_H_12_O_2_	992.6	246.58	1.23794	fruit, apple, banana	116.2
17	Ethyl propanoate	105373	C_5_H_10_O_2_	969.5	240.81	1.32208	fruity, sweet	102.1
18	Ethyl Acetate	141786	C_4_H_8_O_2_	889.2	221.725	1.33779	pineapple	88.1
19	ethyl3-methylbutanoate	108645	C_7_H_14_O_2_	1067.9	278.685	1.65689	fruit	130.2
20	Ethyl2-methylbutanoate-M	7452791	C_7_H_14_O_2_	1048.7	269.956	1.25545	apple	130.2
21	Ethyl butanoate-D	105544	C_6_H_12_O_2_	1037	264.778	1.56217	apple	116.2
22	Methyl acetate	79209	C_3_H_6_O_2_	848.7	212.7	1.19344	——	74.1
24	Ethyl butanoate-M	105544	C_6_H_12_O_2_	1034	263.477	1.20435	apple	116.2
25	ethyl2-methylbutanoate-D	7452791	C_7_H_14_O_2_	1053.6	272.138	1.65246	apple	130.2
26	pentyl formate	638493	C_6_H_12_O_2_	1017.3	256.304	1.61821	——	116.2
27	Ethyl 2-methy lpropionate	97621	C_6_H_12_O_2_	970.9	241.147	1.56588	sweet, rubber	116.2
31	Ethyl formate	109944	C_3_H_6_O_2_	828.6	208.358	1.06969	pungent	74.1
35	Isoamyl acetate-M	123922	C_7_H_14_O_2_	1124.2	310.119	1.31645	banana	130.2
39	Ethyl octanoate-M	106321	C_10_H_20_O_2_	1431.9	674.043	1.49085	fruit, fat	172.3
40	Hexyl acetate	142927	C_8_H_16_O_2_	1272.1	438.223	1.36447	fruit, herb	144.2
	**Alcohols**							
1	2-butoxyethanol	111762	C_6_H_14_O_2_	1453.6	715.276	1.20492	fruity, herbal flavor	118.2
5	3-Methyl-1-butanol	123513	C_5_H_12_O	1204.8	370.638	1.48651	whiskey, malt, burnt	88.1
10	1-hydroxy-2-propanone	116096	C_3_H_6_O_2_	1273.1	439.206	1.23163	pungent, sweet, caramelly, ethereal	74.1
13	2-Methylpropanol	78831	C_4_H_10_O	1086.6	287.413	1.36986	wine, solvent, bitter	74.1
15	2-butanol	78922	C_4_H_10_O	1016.3	255.901	1.34564	wine	74.1
30	Methanol	67561	CH_4_O	891.9	222.355	0.98861	——	32
36	1-pentanol	71410	C_5_H_12_O	1273.4	439.568	1.27064	balsamic	88.1
	**Ketones**							
9	3-Octanone	106683	C_8_H_16_O	1272.6	438.713	1.30156	herb, butter, resin	128.2
29	Acetone	67641	C_3_H_6_O	844.5	211.805	1.11664	fruity	58.1
34	2-Octanone	111137	C_8_H_16_O	1287.2	454.973	1.75587	soap, gasoline	128.2
37	3-Hydroxy-2-butanone	513860	C_4_H_8_O_2_	1293.1	461.642	1.33433	butter, cream	88.1
	**Aldehydes**							
23	(E)-2-butenal	123739	C_4_H_6_O	1053.8	272.274	1.2053	flower	70.1
33	Butanal	123728	C_4_H_8_O	838.2	210.422	1.28146	pungent, green	72.1
	**Terpenoids**							
7	Limonene	138863	C_10_H_16_	1232.4	396.955	1.5777	lemon, orange	136.2
	**Others**							
3	1,3-diethylbenzene	141935	C_10_H_14_	1293.3	461.885	1.20966	——	134.2

**Table 4**: ^a^ RI: Retention Index; ^b^ Rt [sec]: retention time; ^c^ Dt [a.u.]: drift time; ^d^ MW: Molecular Weight; The “*” indicates compounds that were not detected.; ^#^ Odor characteristics were sourced from the Flavornet database, an authoritative online repository for flavor-related information (https://www.flavornet.org/flavornet.html, accessed on 15 November 2024), as well as the following literature: (Mu et al., 2023) [74]; (Xiao et al., 2017) [75]; (Zhang, Tong, et al., 2023) [76]; (Hu et al., 2023) [77]; (Zhou et al., 2022) [69].

### 3.6. Hierarchical Cluster Analysis of Volatile Compounds in Sweet Potato Fermented Brandy Subjected to Diverse Processing Techniques

#### 3.6.1. Dynamic PCA of Samples

The PCA model was constructed using the normalized signal intensities of all identified volatile compounds across the five treatment groups, as extracted from GC-IMS spectra. PCA is a robust tool in multivariate analysis commonly employed to examine complex datasets with multiple quantitative variables [78]. It is widely used in clustering analyses to visualize and interpret high-dimensional data through dimensionality reduction, thereby highlighting patterns and differences among samples [79]. A PCA model is generally considered effective when the combined explanatory power of the first two principal components (PC1 and PC2) exceeds 60% [80].

**Figure 7:**
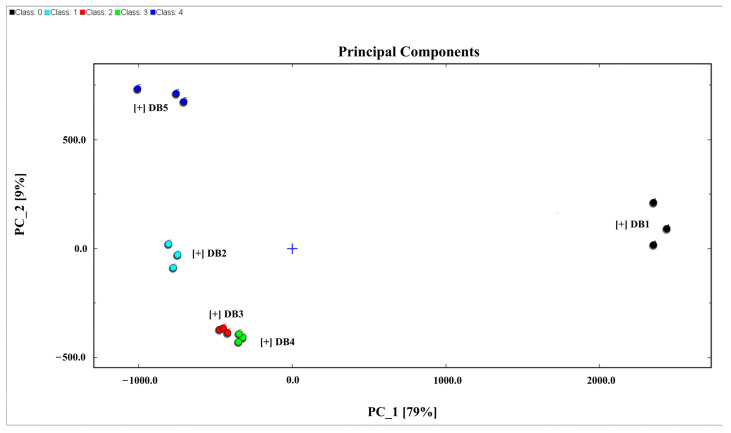
PCA analysis of all samples. DB1: control group; brandy made from raw sweet potatoes. DB2: brandy made from steamed sweet potatoes. DB3: brandy made from boiled sweet potatoes. DB4: brandy made from fried sweet potatoes. DB5: brandy made from baked sweet potatoes.

In this investigation, PCA was used to analyze the volatile compound data from sweet potato brandy samples characterized by their peak positions and intensities via GC-IMS analysis. The data were processed using a dynamic PCA plugin, which clarified the differences among brandies derived from sweet potatoes processed through various methods. As shown in Figure 7, the combined contribution of the first two principal components, PC1 and PC2, was 88%, with PC1 accounting for 79% and PC2 for 9% of the total variance. This far exceeds the 60% benchmark, indicating a robust PCA model. The high variance explained by these components ensures that essential information is retained in the reduced dataset, accurately reflecting the intrinsic differences of the original variables and effectively discriminating between the brandy samples [81].

The PCA plot in Figure 7 spatially distributes samples DB1, DB3, DB4, and DB5 across the quadrants of the Cartesian coordinate system, with DB1 in the first quadrant, DB5 in the second, and both DB3 and DB4 in the third, while DB2 is positioned along the negative x-axis. The clear separation of the samples in the plot underscores the pronounced differences in their characteristics. However, the close proximity of DB3 and DB4 suggests a reduced degree of variance between these two samples compared to the others, indicating more subtle distinctions within the dataset.

**Figure 8:**
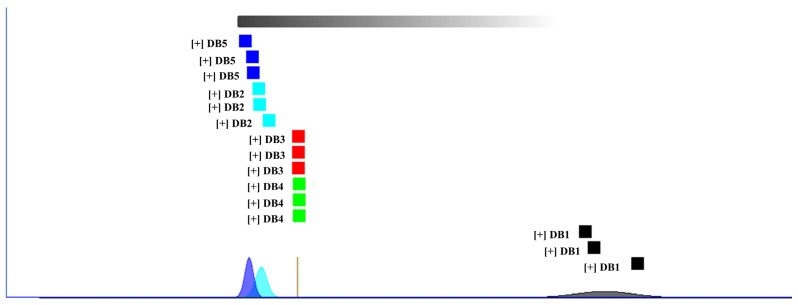
Euclidean distance map between samples. DB1: control group; brandy made from raw sweet potatoes. DB2: brandy made from steamed sweet potatoes. DB3: brandy made from boiled sweet potatoes. DB4: brandy made from fried sweet potatoes. DB5: brandy made from baked sweet potatoes.

Additionally, the Euclidean distances shown in Figure 8 corroborate these findings, revealing that DB3 and DB4 are in close proximity, while DB1 and DB5 are the most distant from each other. This distribution highlights the variations and similarities in the volatile organic compound (VOC) profiles of the brandy samples. Thus, PCA proves to be a powerful analytical tool for distinguishing the unique VOC signatures in brandies made from sweet potatoes subjected to different processing techniques. These systematic comparisons across thermal pretreatments not only reveal the underlying chemical and sensory differences but also offer structured insight for potential product differentiation. While sweet potato brandy is not yet widely commercialized, the current data may serve as a valuable reference point for future industrial applications of root-based distilled beverages.

#### 3.6.2. Fingerprint Similarity Analysis Using Euclidean Distance

Fingerprint similarity analysis utilizes Euclidean distances to assess the comparability of volatile organic compound (VOC) profiles across samples. This method relies on the distance coefficient, with Euclidean distance serving as a key metric for cluster analysis [82]. A larger distance coefficient indicates greater divergence between samples, establishing a direct correlation between the magnitude of the coefficient and the extent of sample dissimilarity. Conversely, a smaller coefficient suggests reduced differences and greater similarity among the samples [83].

As shown in Figure 8, the fingerprint similarity analysis, based on Euclidean distances, corroborates the findings from Figure 6 and Figure 7. The plot clearly distinguishes the brandy samples, with DB1 exhibiting significant separation from DB2, DB3, DB4, and DB5. Notably, the considerable distance between DB1 and DB5, as visualized in the Euclidean distance map (Figure 8), indicates the most pronounced differences, likely due to chemical transformations in sweet potatoes induced by baking, which impart distinct flavor profiles post-fermentation. Although no numerical thresholds were applied to define spectral similarity, the relative distances between samples provide an intuitive and informative indication of VOC divergence in line with exploratory practices commonly employed in GC-IMS-based fingerprint analysis [21].

Furthermore, the close proximity of DB3 and DB4 in Euclidean distance reflects the highest degree of similarity among the sample pairs. This closeness suggests that the VOC profiles of DB3 and DB4 are less influenced by their respective processing methods, highlighting the efficacy of Euclidean distance measurements in detecting subtle distinctions in the sample characteristics.

## 4. Conclusions

This study systematically evaluated the effects of five thermal pretreatments (raw, steamed, boiled, fried, and baked) on the physicochemical parameters, volatile compound composition, and sensory attributes of sweet potato brandy derived from the Yanshu No. 25 cultivar. Sensory evaluation showed that brandies made from raw (DB1) and boiled (DB3) sweet potatoes received the highest overall scores, supported by their more favorable aroma and taste profiles. GC-IMS analysis identified 37 volatile compounds across the samples, with DB1 showing the greatest abundance and diversity of esters and alcohols—compounds known to contribute to fruity and floral notes—whereas DB5 (baked) exhibited increased ketones and methanol levels, accompanied by reduced sensory acceptance. Multivariate statistical analyses (PCA and Euclidean distance clustering) confirmed that the volatile profiles varied significantly among the treatment groups, highlighting the critical impact of thermal processing on flavor development. Taken together, the results suggest that mild or moderate thermal pretreatment can better preserve key aromatic compounds and optimize sensory quality in sweet potato brandy production. These findings provide practical guidance for thermal process selection in the development of high-quality, aroma-rich sweet potato distilled beverages.

## Data Availability

The original contributions presented in the study are included in the article, further inquiries can be directed to the corresponding author.
